# Case Report: Intervention of radiotherapy improves the prognosis of rectal squamous cell carcinoma with high PD-L1 expression and enable patients to obtain NED status

**DOI:** 10.3389/fimmu.2023.1235697

**Published:** 2023-07-14

**Authors:** Fuyin Qu, Linlin Xiao, Yuting Xiao, Chao Gao, Xuan Wang, Yi Wang, Yuanhang Gao, Fengpeng Wu, Ming Liu

**Affiliations:** Department of Radiation Oncology, the Fourth Hospital of Hebei Medical University, Shijiazhuang, Hebei, China

**Keywords:** rectal squamous cell carcinoma, PD-L1, radiotherapy, immunotherapy, chemotherapy

## Abstract

**Background:**

Rectal squamous cell carcinoma (RSCC) is a rare malignancy of the rectal tumor. Due to its extremely low incidence, there is still a lack of high-level treatment evidence and clinical consensus on this disease.

**Case report:**

In this article, we report a treatment process of RSCC with high PD-L1 expression. Firstly, this patient received 2 cycles of Pembrolizumab immunotherapy, but the efficacy was less sanguine. Subsequently, 4 cycles of mFOLFOX6 chemotherapy were synchronously performed on the basis of the initial regimen. Although partial remission was achieved in the lymph nodes thereafter, the changes in the primary lesions were still not significant. After that, the patient received radiotherapy, and followed by 6 cycles of PC (Albumin-binding Paclitaxel and Nedaplatin) regimen chemotherapy combined with Pembrolizumab. Eventually, the patient achieved no evidence of disease (NED) status, and no signs of recurrence or metastasis were found after 12 months of follow-up.

**Conclusion:**

This is the first report of a RSCC patient with high PD-L1 expression achieving a complete response. Looking back over the whole treatment process of this patient, we found that the participation of radiotherapy was the inflection point of prominent efficacy, which may provide a new idea for the selection of comprehensive treatment strategies for patients with RSCC.

## Introduction

Rectal cancer is a common malignant gastrointestinal tumor, among which more than 90% are adenocarcinoma, and squamous cell carcinoma (SCC) only accounts for 0.1-0.25‰ (1). Studies support differences between rectal adenocarcinoma (RAC) and rectal squamous cell carcinoma (RSCC) with respect to epidemiology, pathogenesis, treatment, and prognosis (2). Compared with the more common RAC, RSCC has a worse prognosis (3). RSCC and anal SCC have similar molecular characteristics and are significantly different from RAC (4). The pathogenesis of RSCC is currently unknown and may be associated with smoking, past radiation exposure, squamous dermatitis, chronic proctitis, human immunodeficiency virus (HIV) infection, and human papillomavirus (HPV) infection (5).

Given the rarity of rectal SCC, the clinical data, therapeutic patterns and survival outcomes are mostly limited to individual case reports, pooled meta-analysis and Surveillance, Epidemiology, and End Results (SEER) database analyses (6, 7). Until now, there are no recommendations for the treatment of RSCC. In the past, surgery was the standard treatment (8), and in recent years, chemoradiotherapy (CRT) has become the preferred treatment (9). With the development of medicine, the rise of immunotherapy has brought a new dawn for the treatment of malignant tumors, but the application of immunotherapy in RSCC is still blank. Here, we report a treatment process of a RSCC patient with high PD-L1 expression, and she achieved NED status after individualized comprehensive treatment.

## Case report

**Figure 1:**
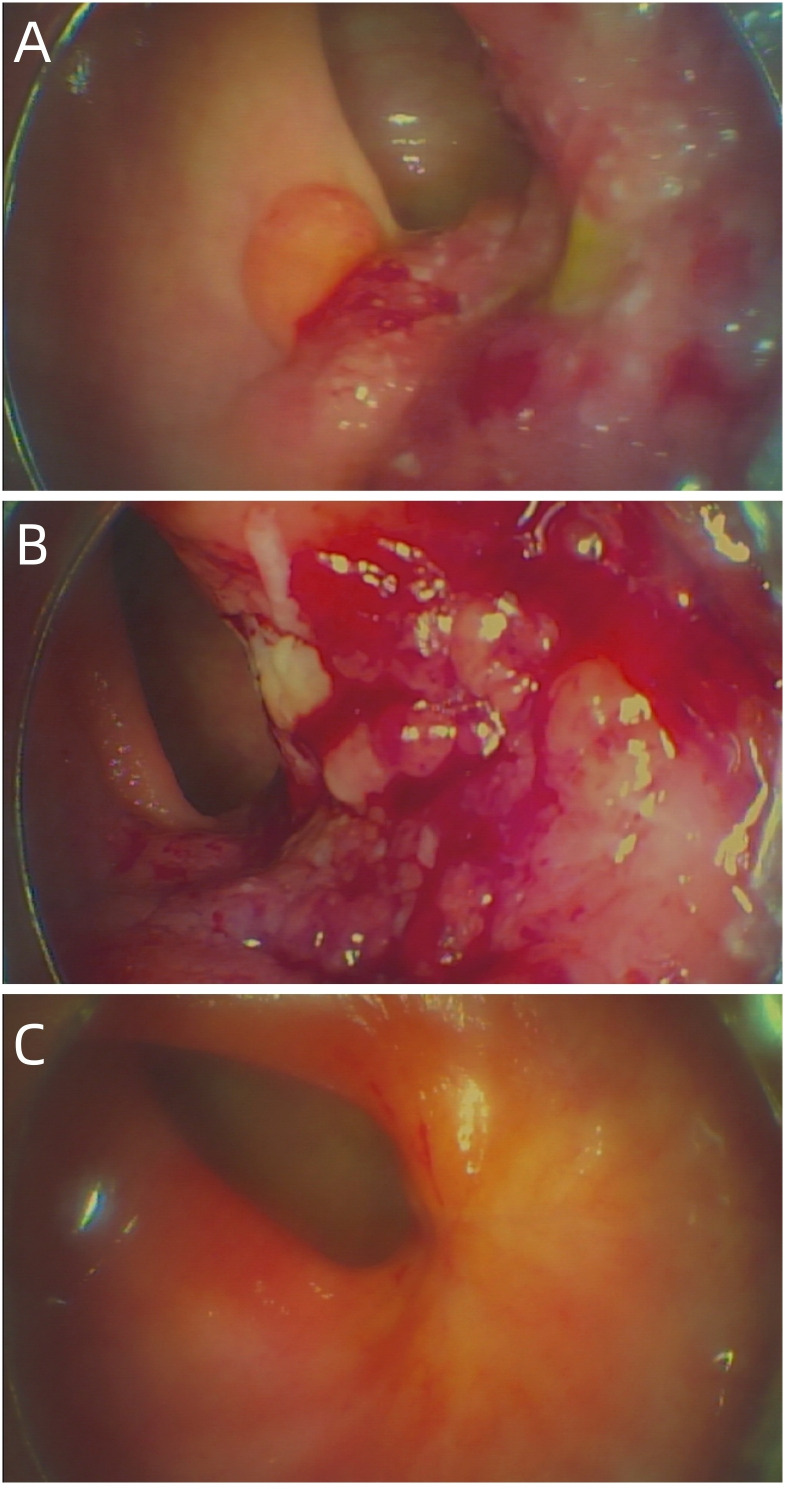
**(A)** The baseline electronic fiber colonoscopy of the patient. **(B)** After 2 cycles of immunotherapy, there was no significant change in the size of the tumor, but the surface was congested. **(C)** Showed that the rectal mass has disappeared and fibrosis can be seen in the area of the original area.

**Figure 2:**
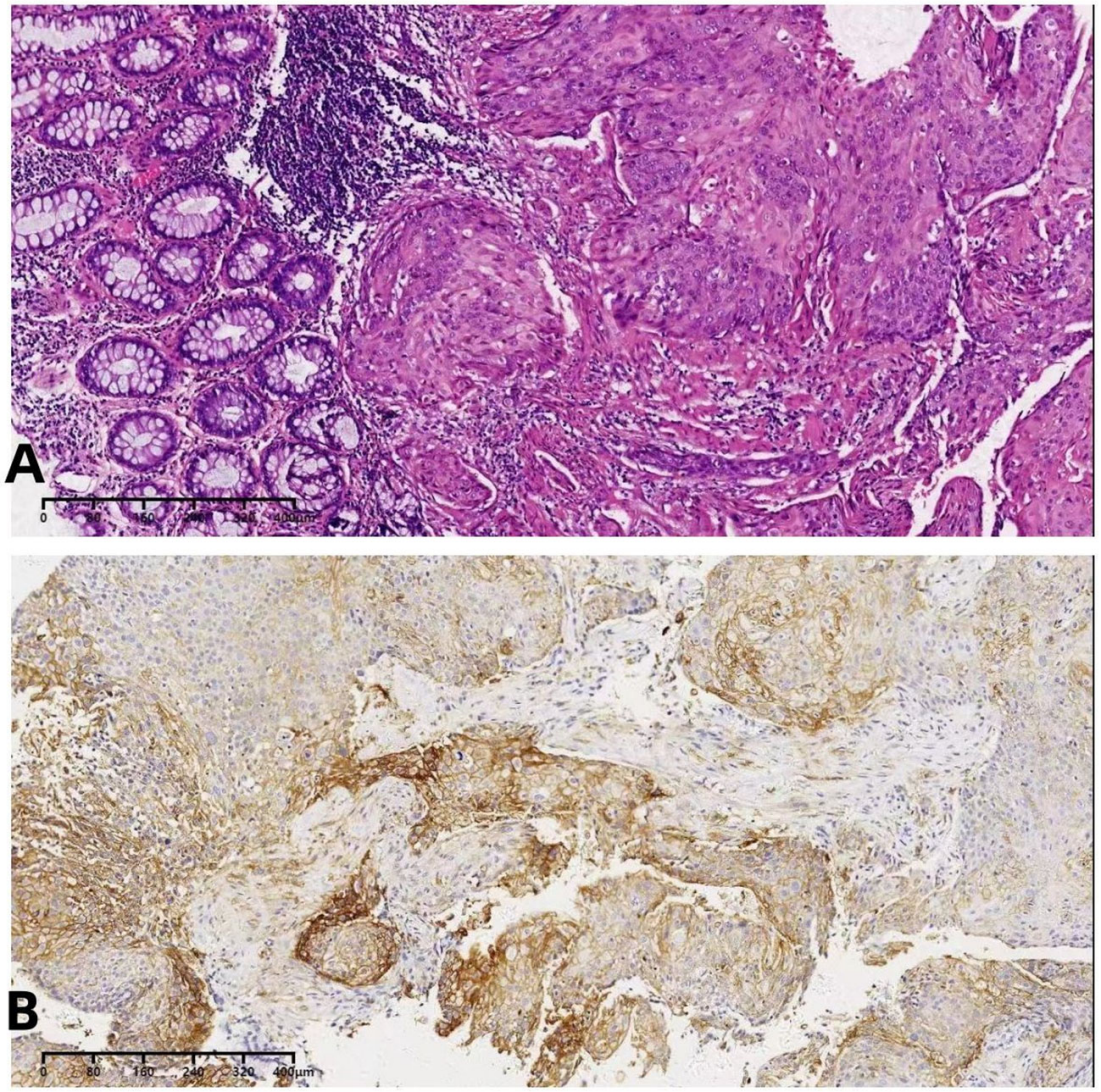
**(A)**The biopsy indicated squamous cell carcinoma. **(B)** The immunohistochemical PD-L1 detection.

**Figure 3:**
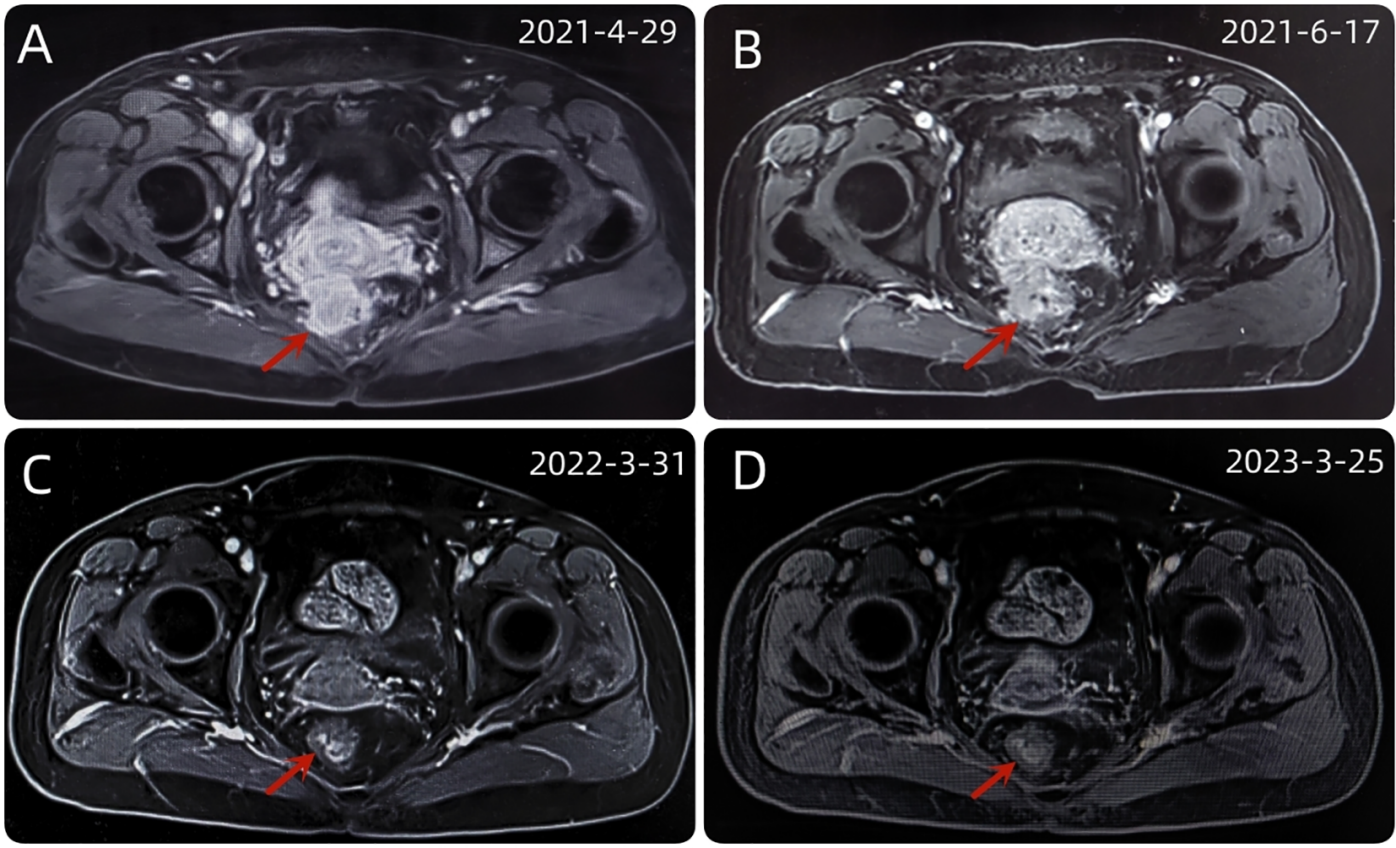
**(A)** The baseline abdominal MRI of the patient. **(B)** After 2 cycles of immunotherapy, the size and shape of the tumor did not change significantly. **(C)** After a series of comprehensive treatment, the original mass disappeared. **(D)** After 1 year of follow-up, the disease was stable and had no recurrence.

**Figure 4:**
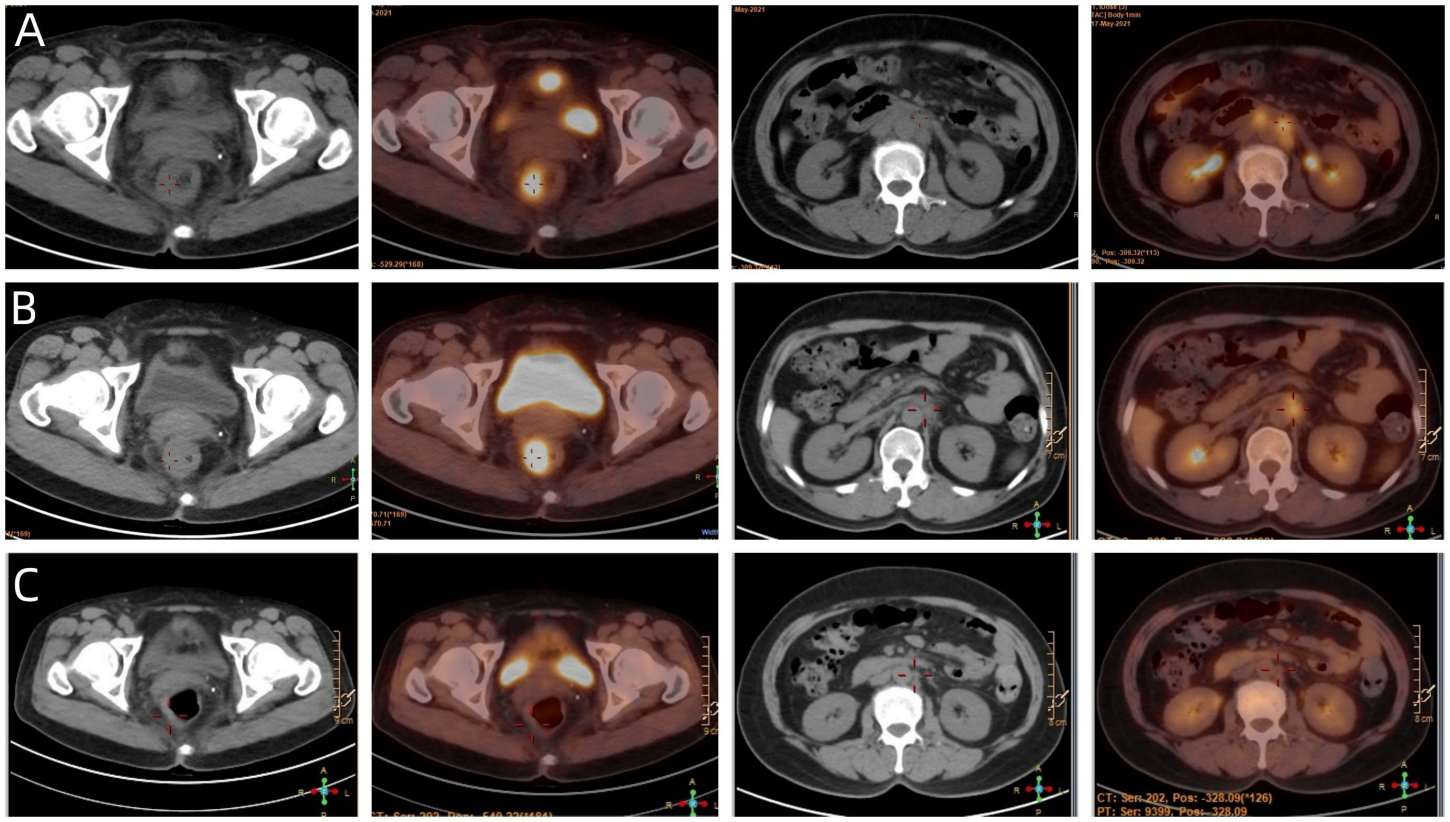
**(A)** The baseline PET/CT of the patient. **(B)** Showed that most of the abnormal lymph nodes founded on the baseline scan had disappeared, but the rectal mass and para-aortic lymph nodes at the level of the second lumbar spine has not changed significantly. **(C)** Showed that the patient achieved NED status eventually.

A 54-year-old female patient was admitted to the outpatient department of Abdominal Radiotherapy of our hospital due to intermittent stool bleeding in April 2021. Physical examination found that the appearance of the anus was normal, a mass could be touched in the site of 5 cm from the anus (6-12 o’clock) during digital rectal examination, the anal sphincter contracted normally, and the gloves were slightly stained with blood after the fingers were removed. Electronic fiber colonoscopy showed that the rectal mucosa was rough, the tumor protruding into the rectum, 5-10 cm from the anus, and the lower pole of the mass was located 1cm above the dentate line (**Figure 1A**). Biopsy of the lesion is SCC (**Figure 2A**). The results of immunohistochemistry showed that AE1/AE3 (+), P40 (+), CK5/6 (+), Ki-67 (70%), MLH1 (+), FMS2 (+), MSH2 (+), MSH6 (+), PD-L1 (VENTANA SP263) (CPS:60), PD-L1 (DAKO 22C3) (CPS:50) (**Figure 2B**). Gene detection showed that no mutation sites were found in KRAS, NRAS and BRAF and Microsatellite-instability (MSI) status was microsatellite stable (MSS). Abdominal MRI found that carcinoma invaded the posterior wall of the uterus, and the distance between the distal end of the tumor and the anal margin was 6.3 cm (**Figure 3A**). The SUVmax of primary lesions was 13.8, and of lymph nodes of mesentery, iliac vessels, abdominal aorta, mediastinum, left neck and bilateral supraclavicular were all above 2.0 in ^18^F-FDG PET/CT scanning. Of these suspected lymph nodes, para-aortic lymph nodes at the level of the second lumbar spine had the strongest uptake capacity for ^18^F-FDG, with a SUVmax of 8.0 (**Figure 4A**).

### Anti-tumor therapy

Up to now, there is no effective treatment strategy recommended for patients with RSCC. This patient has a particularity, that is, her husband is an oncologist in our center, and he participated in the formulation of the initial treatment plan with great weight. Considering the high expression level of PD-L1, immune checkpoint inhibitors (ICIs) was selected as the initial treatment. After received 2 cycles of Pembrolizumab immunotherapy, the patient’s symptoms did not significantly relieve and the size of the tumor has not shrunk in imaging (**Figure 3B**). Re-examination of electronic sigmoidoscopy revealed a protuberant mass 5-10cm from the anus, with surface congestion (**Figure 1B**). In view of the above, and the fact that no new suspicious lesions were found in the reexamination imaging, we assessed the efficacy at this stage as stable disease (SD).

Given the less sanguine efficacy of the application of single pembrolizumab, a discussion was conducted by the multidisciplinary team (MDT) (Incl. Medical imaging specialist, Gastrointestinal surgeons, Gastroenterologists, Oncologists, Pathologists, Radiotherapy specialist, et al.). In view of the less sanguine efficacy of this patient after 2 cycles of PD-1 inhibitor, the treatment was adjusted to pembrolizumab combined with mFOLFOX6 (fluorouracil, leucovorin, and oxaliplatin) for 4 cycles from June 21 to September 29, 2021. The patient’s compliance was good during the whole treatment process, and the adverse reactions were mild. Laboratory tests showed no obvious hematological toxicity and the monitoring of cardiac function, pituitary function, BNP, troponin, and thyroid function showed no abnormal changes. After this course of treatment, the symptoms of blooding in stool basically disappeared, but tenesmus still exist. A second PET/CT was performed, at which the SUVmax of the primary lesion was 10.2. Although most of the abnormal lymph nodes founded on the baseline scan had disappeared, the para-aortic lymph nodes at the level of the second lumbar spine has not changed and with SUVmax of 4.1 (**Figure 4B**).

Therefore, at the time of the second MDT, experts agree that most of the metastatic lymph nodes have disappeared, and the remaining lesions are mainly concentrated in the primary lesions and paraaortic lymph nodes. Next, local treatment (Incl. surgical resection, radiotherapy) should be strongly recommended. Since the patient refused to operate, we performed pelvic IMRT (PTV: 50.4Gy/28F, 1.8Gy/F) and abdominal lymph nodes SBRT (PTV: 42Gy/7F, 6Gy/F). The radiotherapy process was smooth and the patient had no obvious discomfort. The findings of efficacy evaluation of 2 weeks after the end of radiotherapy showed that the symptoms of tenesmus and blooding in stool disappeared, the lesion was significantly smaller, and no new suspicious lesions were found. we assessed the efficacy at this stage as partial response (PR).

Considering that the therapeutic effect of mFOLFOX6 to this patient is not optimistic, the third MDT was carried out, and then the chemotherapy regimen was changed to PC (Albumin-binding Paclitaxel and Nedaplatin), which is effective in the treatment of esophageal, lung and other SCC, and pembrolizumab was continued. A total of 6 cycles of this regimen were performed from November 13, 2021 to March 25, 2022. The treatment process was smooth and the patient showed no significant blood toxicity or liver and kidney function damage. At last, there was only mild numbness in the feet without pain and other discomfort, which was considered as neurotoxicity of the chemotherapy drugs. After this stage treatment, the patient’s efficacy was evaluated in detail. Re-examination by electronic sigmoidoscopy showed that the tumor was 5cm from the anus with scar changes after radiotherapy, the mucosa was smooth, and no obvious new organisms were observed (**Figure 1C**). Colonoscopy showed that the rectal mass disappeared and the intestinal mucosa showed scar changes. No occupying lesions were found in abdominal and pelvic MRI scans (**Figure 3C**). PET/CT showed mild uptake of ^18^F-FDG at the primary tumor site with SUVmax of 1.6, and all abnormal uptake of ^18^F-FDG in lymph nodes seen at baseline scans disappeared (**Figure 4C**). Within 12 months after the end of the overall treatment regimens, four follow-up visits for the patient were conducted and no recurrence lesions were found (**Figure 3D**). Therefore, we believe that this patient has obtained NED status.

## Discussion

RSCC is a rare gastrointestinal malignancy, and there is still a lack of high-level treatment evidence and clinical consensus on this disease (9). The specificity of this patient lies in its high PD-L1 expression level and MSS status. Takashi Kojima et al. found that in patients with esophageal SCC with positive PD-L1 expression, pembrolizumab reduced the risk of disease death by 37% compared with chemotherapy alone, and the 1-year OS was nearly doubled (10.3m *vs.* 6.7m) (10); In the first-line treatment of advanced squamous lung cancer, pembrolizumab combined with PC regimen significantly increased median PFS (6.4m *vs.* 4.8m) and OS (15.9m *vs.* 11.3m) compared with chemotherapy alone (11). However, for colorectal cancer in KEYNOTE-028 study (12), only 1 of 23 patients with PD-L1 positive expression obtained the therapeutic response of pembrolizumab, and this was MSI-H case. For our patient, her husband strongly requested ICIs based on the high expression of PD-L1, so pembrolizumab was selected as the initial treatment regimen. Unfortunately, the efficacy of this stage of treatment is not obvious, we considered that it may be related to the treatment heterogeneity caused by the special location of the tumor.

As is known to all, due to its special location and function, the colorectal is the main habitat organ of human flora, and its complex microenvironment plays an important role in the occurrence and development of various tumors (13). Studies have found that the status of intestinal flora is closely related to the responsiveness of tumor patients to immunotherapy. Gopalakrishnan et al. tested the oral and intestinal microbes of 112 melanoma patients receiving anti-PD-1 immunotherapy and found there were significant differences in the diversity and composition of intestinal microbiome between patients who responded and those who did not (14). Routy B (15) found that the main resistance to ICIs in tumor patients could be attributed to abnormal intestinal microbial composition. Researchers transplanted fecal microbiota of cancer patients responding to ICIs into sterile or antibiotic-treated mice, which could improve the anti-tumor effect of ICIs.

In view of the less sanguine efficacy of this patient after 2 cycles of PD-1 inhibitor, we combined the classic mFOLFOX6 chemotherapy regimen for colorectal cancer on the basis of the original regimen. After 4 cycles of this treatment, the ^18^F-FDG metabolic capacity of the positive lymph nodes was significantly reduced, but the size and metabolic capacity of the primary focus were not significantly improved. In combination with the above literature, we consider that this situation may also be related to the large difference in the microflora environment of the primary lesion located in the intestine and that of the lymph nodes in the non-intestinal area.

As a local treatment, radiotherapy has been widely used in the comprehensive treatment of middle and low locally advanced rectal cancer. A SEER database analysis of 999 patients with RSCC treated between 1998 and 2011 showed that the median OS was significantly higher in patients who received radiotherapy than in those who did not (135m *vs.* 51m) (7). Similarly, in a National Cancer Database (NCDB) analysis of 3405 RSCC patients between 2004 and 2015, people who received definitive chemoradiation only had a higher median OS as compared to that of surgery alone (108m *vs.*76m) (p=0.012) (16). Although currently there is no unified conclusion on the radiotherapy target area, irradiation dose and segmentation method of RSCC, our patient obtained a large degree of PR after receiving conventional and large segmentation radiotherapy in pelvic and abdominal lymph nodes, which further verified the important value of radiotherapy in RSCC.

A large number of studies have shown that radiotherapy plays an important role in improving the efficacy of immunotherapy in tumor patients, which is related to the anti-cancer mechanism that can benefit from each other between the two treatments. Gao et al. previously reported a case of esophageal SCC patient, who achieved good efficacy when anti-PD-1 immunotherapy was followed by three-dimensional conformational radiotherapy for 10 times (TD:20Gy), and the lesions of the patient’s esophagus and mediastinal were significantly reduced (17). In addition, immunotherapy is revolutionizing existing treatment strategies in the routine treatment of renal-cell carcinoma. Novel fractionation schedules of radiotherapy, consisting of high doses in few fractions, can overcome the radioresistance of this tumor. This effect mediated by the immune system can be enhanced associating radiotherapy with immunotherapy (18).Studies have found that radiation can directly damage the DNA of tumor cells, stimulate the emergence of new antigens (19), up-regulate the expression levels of chemokines CXCL10 and CXCL16 in the tumor microenvironment, promote the migration of Cytotoxic T lymphocytes to tumors (20, 21), and enhance the expression of PD-L1 on the surface of target cells, and improve the effect of anti-PD-L1 treatment (22, 23). In addition, PD-L1 expression by dendritic cells is a key regulator of T-cell immunity in cancer (24) and local high-dose irradiation results in activation of tumor-associated dendritic cells that in turn support tumor-specific effector CD8+ T cells, thus identifying the mechanism that underlies radiotherapy-induced mobilization of tumor specific immunity (25). In addition to radiotherapy, chemotherapy can also have a good synergistic effect with immunotherapy. Chemotherapy can destroy the activity of immunosuppressive cells, such as regulatory T cells (Treg), interleukin-17-secreting T cells, myeloid-derived suppressor cells (MDSC), and tumor-associated macrophages (TAM). Not only that, chemotherapy can also promote the immune response by inducing tumor cell apoptosis, up-regulation of MHC class 1 molecules expression, and dendritic cell maturation (26, 27). In conclusion, chemoradiotherapy can improve the efficacy of immunotherapy. In the early treatment of this patient, although the efficacy of pembrolizumab was not obvious, we continued to apply pembrolizumab after radiotherapy, and adjusted the chemotherapy regimen from mFOLFOX6 to PC. The NED status was obtained after 6 cycles of PC combined with ICIs regimen, and no signs of recurrence or metastasis were observed after 12 months of follow-up.

In the treatment process of this patient, there were some limitations. To begin with, our initial treatment scheme was interfered by non-medical factors. In fact, the standard medical behavior in some areas is greatly influenced by the opinions of patients’ families. This is not only a medical problem, but also a cognitive problem of humanities. It is a long way to go to overcome this phenomenon. Another limitation is that we have too high expectations for the high expression of PD-L1 in SCC and have not fully considered the specificity of ICIs in the treatment of colorectal cancer. The final limitation lies in the choice of chemotherapy regimen. We conservatively adhere to the classic chemotherapy regimen of colorectal cancer, without in-depth discussion of its special pathological types. Despite these setbacks in the whole treatment process, the efficacy of patients has been significantly improved after radiotherapy. It can be said that the choice of radiotherapy is an important inflection point for the improvement of the patient’s efficacy, and the adjustment of the system treatment scheme after radiotherapy further consolidated and improved the efficacy.

In conclusion, for RSCC patients with high PD-L1 expression and MSS status, the application of single ICIs seems not to be a favorable treatment option. Comprehensive treatment with radiotherapy may be a therapeutic strategy that can benefit such patients. However, the optimal time of radiotherapy intervention and the application details of radiotherapy technology are still outstanding issues. It is necessary to accumulate and summarize the treatment experience of this rare disease and carry out relevant clinical trials.

## Data availability statement

The original contributions presented in the study are included in the article/supplementary material. Further inquiries can be directed to the corresponding author.

## Ethics statement

Written informed consent was obtained from the individual(s) for the publication of any potentially identifiable images or data included in this article. Written informed consent was obtained from the participant/patient(s) for the publication of this case report.

## Author contributions

Conceptualization, ML, FW. Data curation, FQ, LX, YX. Formal analysis, XW and CG. Resources, YG, YW. Validation, FQ, ML. Writing-original draft, FQ, LX. Writing-review and editing, ML. All authors contributed to the article and approved the submitted version.
